# Cost-effectiveness of postural exercise therapy versus physiotherapy in computer screen-workers with early non-specific work-related upper limb disorders (WRULD); a randomized controlled trial

**DOI:** 10.1186/1745-6215-10-103

**Published:** 2009-11-17

**Authors:** Marjon D van Eijsden, Sylvia A Gerhards, Rob A de Bie, Johan L Severens

**Affiliations:** 1 Department of Physical Medicine and Rehabilitation, University Hospital Maastricht, Maastricht, the Netherlands; 2 Department of Clinical Psychological Science, Faculty of Psychology, Maastricht University, Maastricht, the Netherlands; 3 Department of Epidemiology & Caphri Research Institute, Maastricht University, Maastricht, the Netherlands; 4 Department of Health Organization, Policy and Economics, Faculty of Health Sciences & Caphri Research Institute, Maastricht University, Maastricht, the Netherlands; 5 Department of Clinical Epidemiology and MTA, University Hospital Maastricht, Maastricht, the Netherlands

## Abstract

**Background:**

Exercise therapies generate substantial costs in computer workers with non-specific work-related upper limb disorders (WRULD).

**Aims:**

To study if postural exercise therapy is cost-effective compared to regular physiotherapy in screen-workers with early complaints, both from health care and societal perspective.

**Methods:**

Prospective randomized trial including cost-effectiveness analysis; one year follow-up. Participants: Eighty-eight screen-workers with early non-specific WRULD; six drop-outs. Interventions: A ten week postural exercise program versus regular physiotherapy. Outcome measures: Effectiveness measures: Pain: visual analogous scale (VAS), self-perceived WRULD (yes/no). Functional outcome: Disabilities of Arm, Shoulder and Hand- Dutch Language Version (DASH-DLV). Quality of life outcome: EQ-5D.

Economic measures: health care costs including patient and family costs and productivity costs resulting in societal costs. Cost-effectiveness measures: health care costs and societal costs related to the effectiveness measures. Outcome measures were assessed at baseline; three, six and twelve months after baseline.

**Results:**

At baseline both groups were comparable for baseline characteristics except scores on the Pain Catastrophizing Scale and comparable for costs. No significant differences between the groups concerning effectiveness at one year follow-up were found. Effectiveness scores slightly improved over time. After one year 55% of participants were free of complaints. After one year the postural exercise group had higher mean total health care costs, but lower productivity costs compared to the physiotherapy group. Mean societal costs after one year (therefore) were in favor of postural exercise therapy [- €622; 95% CI -2087; +590)]. After one year, only self- perceived WRULD seemed to result in acceptable cost-effectiveness of the postural exercise strategy over physiotherapy; however the probability of acceptable cost-effectiveness did not exceed 60%.

Considering societal costs related to QALYs, postural exercise therapy had a probability of over 80% to be cost-effective over a wide range of cost-effectiveness ceiling ratios; however based on a marginal QALY-difference of 0.1 over a 12 month time frame.

**Conclusion:**

Although our trial failed to find significant differences in VAS, QALYs and ICERs based on VAS and QALYs at one-year follow-up, CEACs suggest that postural exercise therapy according to Mensendieck/Cesar has a higher probability of being cost-effective compared to regular physiotherapy; however further research is required.

**Trial registration:**

ISRCTN 15872455

## Background

The prevalence of Work-Related Upper Limb Disorders (WRULD) in the Dutch working population is estimated about 19-30% [1]. Due to expectations of increasing intensity of computer screen-work, the prevalence of WRULD among screen-workers is expected to increase even more [2,3].

WRULD can result in decreased productivity, increased medical consumption and consequently increased costs. A recent study estimates the total yearly costs due to specific and non-specific WRULD in the Netherlands at about 2.1 billion Euros, consisting of medical costs, costs due to decreased productivity, absenteeism related to WRULD and disability pensions [4].

It can be assumed that WRULD is associated with a decreased quality of life [5].

Of all WRULD complaints, it is estimated that specific disorders are responsible for about 13-37% of them. The majority concerns non-specific WRULD [1,2].

In the Netherlands non-specific WRULD is treated within various medical and paramedical disciplines [1]. Postural exercise (PE) therapy according to Mensendieck/Cesar [6] and regular physiotherapy (RP) are two treatments in the Netherlands used for patients suffering from WRULD [7].

Very little reliable research is available regarding the effectiveness of exercise and other treatments in non-specific WRULD [8-11].

The same goes for cost-effectiveness studies [12,13] in which the quality of life rarely was used as an outcome measure in musculoskeletal disorders [14].

The high prevalence, costs and decreased quality of life signify a large impact of non-specific WRULD.

Beyond effectiveness studies, cost-effectiveness studies in relation with quality of life are needed in patients with non-specific WRULD to be able to improve health care and to lower the costs.

As the department of rehabilitation of the Maastricht University Hospital acts as a tertiary referral centre for non-specific WRULD complaints a cost-effectiveness study among computer screen-workers with early stages of non-specific WRULD was set up.

In the current study we tested if postural exercise therapy according to Mensendieck/Cesar is cost-effective with respect to pain, disability and quality of life as when compared to regular physiotherapy in computer screen-workers with early stages of non-specific WRULD, both from a health care and societal perspective.

## Methods

### Design

A prospective randomized clinical trial was set up among computer screen-workers with early non-specific work-related upper limb disorders [11]. Recruitment took place by advertisement in local newspapers, by personal contact with occupational physicians of large industries and by mailing to general practitioners in South Limburg. Screen-workers fulfilling the inclusion criteria were invited to take part in this study. Selection and diagnosis were performed by an independent occupational physician familiar with the diagnosis of "non-specific WRULD" who was blinded to allocation sequence.

Within two weeks after eligible patients were selected and invited to participate by the occupational physician, baseline assessments were performed at one of two locations, either the Maastricht University Hospital or the Institute for Rehabilitation Research in Hoensbroek, a small town in the south-eastern region of the Netherlands.

Participants were randomized to the PE group or the RP group in strata depending on the duration of the complaints (cut-off point six weeks). Blocks of four were generated for each stratum by means of a computer generated random sequence table.

Randomization was concealed because a research assistant, who was not involved in the selection of the participants, allocated participants to groups using a list of random numbers which was generated before commencement of the trial. Because both interventions were active, blinding of participants and therapists was not possible. In both groups, the ten week intervention started within one week after baseline measures were completed. Outcome measures were collected at baseline and at three, six and twelve months where the same questionnaires were completed using a computer under the supervision of a research assistant. The research assistant instructed participants about the questionnaires, which had to be completed by using a computer in the participant's usual manner. The computer workstation was custom-made for this purpose for each participant. Only the pain outcome measure was assessed by the participants filling in the forms by pen during four sequential working days [15]. Although the research assistant was blinded to group allocation, all outcome measures were self-reports so they were not blind. The completion of the questionnaires by the participants took approximately one hour each time.

This research project was approved by the Medical Ethical Committee of the University Hospital of Maastricht.

### Participants

Computer screen-workers with early non-specific WRULD [11]. Early non-specific WRULD were defined as pains and tingles in upper back, neck, shoulders, arms or hands related and restricted to computer screen-work, not yet present during other daily activities and not labelled as a specific diagnosis such as tennis elbow. Computer screen-workers were defined as those employees performing computer work, with or without the use of a mouse, for at least twenty hours per week and at least four hours continuously per day. Computer screen-workers were chosen because they represent a homogeneous group who are at risk for developing non-specific WRULD [2,16].

To be eligible for this study, participants had to fulfil the following inclusion criteria:

- were computer screen-worker at the time of first complaints and being employed in present job for at least three months

- had non-specific WRULD with symptoms existing longer than two weeks but shorter than three months

- aged between 20 and 45 years

Excluded were patients not fulfilling the inclusion criteria and patients with non-specific WRULD during other daily activities such as teeth brushing and car driving, patients with specific WRULD (e.g. carpal tunnel syndrome, tennis elbow, golfers elbow, tendonitis de Quervain), patients with other diseases of musculoskeletal system (e.g. fibromyalgia, hyper mobility syndromes), pregnant patients, patients who were on sick leave and patients who already had received therapy for their complaints or who had received postural exercise therapy during the last five years.

### Interventions

One group of participants received PE therapy, in the Netherlands known as Mensendieck and Cesar. PE therapy according to Bess Mensendieck and Maria Cesar do not differ basically and both therapies and their training programs have been assimilated since the fusion of both societies in 2004 [6,11]. PE therapy according to Mensendieck/Cesar is in use in the Netherlands, the Scandinavian countries and France. PE therapy promotes a method of body posture- and movement education by exercises in which the integration of body and mind takes place in order to improve consciously poor body posture and bad movement habits in relation to daily life activities. The core of the therapy is to make use of feedback from muscle-, joint-, tendon- and ligament positions by means of audio- (verbal instructions), visual (mirrors and video records) and proprioceptive registered signals [6]. It is hypothesized that this feedback, repeatedly offered to and transformed in the central nervous system, will lead in the long term to automatic improvement of postural and movement habits with generalization to daily activities aiming at decrease of complaints. Training in patient specific daily life activities such as computer work forms a part of this therapy. The four therapists involved in this study were trained in treating patients with non-specific WRULD.

The other group of participants received RP and was treated by four physiotherapists who attended a WRULD-course. They did not make use of applications or massage techniques. Active muscle training and fitness exercises were part of the therapy. The focus was on improvement of muscle condition for long-lasting static postures.

All participants in both treatment arms received ten weeks of therapy according to protocol [11].

The PE group received in total one and a half hours more therapy compared to the RP group, although the last group received six more sessions (Table 1).

**Table 1:** Therapy schedules

Weeks	Postural Exercise therapy (PE) Per week	Regular Physiotherapy (RP) Per week
1-3	2 × 1 hour	3 × 1/2 hour

4-6	1 × 1 hour	2 × 1/2 hour

7-8	1 × 1/2 hour	1 × 1/2 hour

9	Exercises at home	Exercises at home

10	Final session 1/2 hour	Final session 1/2 hour

Total hours treatment	10 1/2 hours	9 hours

Treatments were paid for by health care insurance companies.

### Outcome measures

#### Baseline characteristics

At baseline, besides the effectiveness measures (see further) sex, age, number of working hours and level of education were assessed.

Participants were labeled as "highly educated" if they had at least a bachelor's degree.

As this syndrome is related to work, the number of working hours was registered. Because onset and course of non-specific WRULD are influenced by physical, psychosocial and personal risk factors, [2,16-18] variables assessing these risk factors were measured at baseline.

The following variables were assessed:

1. The validated Groningen Fitness Questionnaire [19] was used to measure individual self-reported fitness level.

2. The Dutch version of the Job Stress Survey (JSS) [20] was used to measure job stress experienced at the work place.

3. The Dutch version of the Multidimensional Perfectionism Scale of Frost (MPS-F) measures (neurotic) perfectionism [17,21].

4. The Dutch version of the State-Trait Anxiety Inventory (STAI) measures state-respectively trait anxiety [22]

5. The Dutch version of the Pain Catastrophizing Scale (PCS) has been used to measure to which extend people who suffer from pain experience catastrophizing thoughts [23].

### Effectiveness measures

As a primary outcome measure we used the horizontal numerical visual analogous ten cm scale (VAS) according to Jensen [15] to measure pain at baseline and pain in course of time at the location with the highest pain intensity. In our research project pain was measured at each measurement moment by the participants themselves by hand during four sequential working days/four fixed times a day (at 11, 14, 17 and 20 o' clock) to get a relevant impression about the existence of pain during the whole working week. The final VAS outcome measure was recalculated as the average of sixteen ratings over the four days. In addition, self-perceived WRULD at each follow-up moment was assessed by a dichotomous variable which was the answer to the question: "do you still perceive non-specific WRULD complaints, yes or no?"

As a secondary outcome measure we used the Disabilities of the Arm, Shoulder and Hand- Dutch Language Version (DASH-DLV) questionnaire [24] to measure physical function and symptoms and the disabilities to fulfil daily life activities. At least 27 out of 30 items must be completed to calculate a score from 0 till 100. A lower score indicates a lower disability rate.

To measure the generic quality of life we made use of the EQ-5D of the EuroQol Group [5,25]. This questionnaire is in use in cost-effectiveness studies [13]. The questionnaire consists of five questions regarding the dimensions mobility, self-care, usual activity, pain/discomfort and anxiety/depression. Each question has three response categories ranging from no problem, some problems and many problems. Using a standardised algorithm, the end score of the EQ-5D is a utility, falling within a value scale of zero (dead) to one (perfect health) [5,13,26]. The EQ-5D is used to calculate the quality adjusted life year (QALY) [13,26]. The QALY was corrected for differences in baseline utility using regression-correction. A regression analysis was performed with the utility-score during the follow-up measurement as the dependent variable and the baseline utility-score as independent variable. For correction, the Beta of this equation is multiplied with the individual baseline utility-score.

All scales are commonly used internationally and are reliable and validated.

#### Economic outcome measures

Costs are defined from the societal perspective. These are subdivided into health care costs (including out of pocket costs for the patient and family) and productivity costs. Only costs related to WRULD are included in the analyses. Costs are determined by multiplying the volume reported on each cost item by the estimated costs per unit (Table 2). Out of pocket costs for patient and family were directly measured in the payment. A questionnaire measuring health care costs and costs for patient and family has been used. Missing items in this questionnaire are interpreted as being zero if there simultaneously were markings in cost items elsewhere in the cost questionnaire. The health care costs comprise items like GP visits, home care, medication etc. The costs for the patient and family consist of the reported devices and domestic home care.

**Table 2:** Standard cost prices

Unit	Standard cost price (euro), index 2005			References
Productivity loss per hour	36.00 per hour			Oostenbrink et al., 2004

General Practitioner				
- Consultation *	30.14 per consultation			Oostenbrink et al., 2004
- Visit at home *	60.29 per consultation			Oostenbrink et al., 2004
- Contact by phone *	15.07 per consultation			Oostenbrink et al., 2004
- Repeat prescription *	15.07 per consultation			Oostenbrink et al., 2004
- Assistant *	15.07 per consultation			Proxy: 1/2 standard price GP consultation

Cesar/Mensendieck treatment (PE)*	34.32 per session			Oostenbrink et al., 2004

Physiotherapy treatment (RP)*	33.95 per session			Oostenbrink et al., 2004

Day treatment	235.69 per session			Oostenbrink et al., 2004

Home care (domestic and alpha help) *	32.38 per hour			Oostenbrink et al., 2004

Ergo therapy *	38.48 per session (30 min. in institution)			Dutch Department for Ergo therapy, personal communication (phone call), 24.03.2006

	*Min.*	*Max.*	*Mean*	

Polyclinic consultation (radiology, orthopedics, specialist in general)	57.64	102.92	80.28 per consultation (min. 10 min in a general hospital, max. 15 min in an academic hospital)	Oostenbrink et al., 2004

Psychology in primary health care	64.36	88.12	79.07 per consultation (45-50 min.)	Mean tariff of diverse psychologist practices in primary health care

Company (occupational) doctor	123.76	183.17	153.47 per consultation (60 min.)	"Nederlandse Vereniging voor Arbeids- en Bedrijfsgeneeskunde", personal communication (phone call), 2006

Medication			per box	CvZ, 2006 ^30^
- Aleve (Naproxen, 220 mg, 20 tablets)	2.71	4.26	3.48	
- Ibuprofen (400 mg, 20 tablets)	1.65	8.43	5.04	
- Ibuprofen (600 mg, 20 tablets)	8.32	8.8	8.56	
- Ibuprofen (400 mg, 50 pieces tablets/coated tablet)	4.13/4.46	11.34/12.54	8.12	
- Diclofenac (mean of different mg, 30 tablets)	6.76	15.47	10.7	

Devices			Per device	Ansil company, personal communication (mail), 2006
- Optical mouse	21.78	39.6	30.69	
- Ergonomic mouse	44.55	54.46	49.51	
- Pen mouse tablet	127.72	127.72	127.72	
- Ordinary/wireless mouse	5.94	13.86	9.9	
- Document holder	56.44	87.19	71.82	
- Desk chair	445.54	990.1	717.82	
- Keyboard	34.65	48.51	41.58	
- Bureau adjustable in height (electric)	485.15	1188.19	836.67	
- Workplace screening	282.18	475.25	378.72	University of Maastricht, department "Arbo & Milieu", personal communication: mail, 29.05.2006

* On the original cost price a surcharge of 45% overhead and accommodation costs is calculated.

The number of PE and RP sessions during the ten week intervention period registered by the therapists is used to calculate the health care costs of intervention sessions during this period.

Costs concerning productivity loss are based on the reported sick leave from work due to non-specific WRULD. Data concerning absenteeism are collected in a questionnaire concerning employment and absence through illness [27]. Productivity costs are calculated according to the friction-cost method, indicating that almost everyone is replaceable in the labour process [28]. A friction cost period of 22 weeks or 154 days is adopted [28].

In the cost calculation one general cost price per lost hour of productivity is used for all patients. The number of days absent from work is related to the number of working days and working hours reported by the individual patient. Economic data are gathered three times during the one year follow-up period by the questionnaires, each time measuring the last two months prior to the questionnaires. These assessments took place at the same time as the effectiveness measures. The costs are based on these questionnaires and extrapolated to the costs during the full one year follow-up period.

The handbook of Oostenbrink et al [29] is used as a guideline for determining the cost prices.

Those cost prices of health care services not mentioned, being ergo therapy, psychology and care by occupational doctor are obtained from professional organizations. Prices of medication are obtained from the Dutch College of Health Insurance [30].

Cost prices of devices are obtained from the reporting of patients. If the cost price of a relevant device is not reported by the patient, a suitable minimum, maximum and mean cost price is estimated. Cost price estimates of devices are obtained from an organization specialized in devices for ergonomic work places. For some of the cost items there was one mean cost price, for other items a mean cost price is calculated based on a minimum and maximum cost price. All cost prices are indexed to 2005 by using the price index numbers of the Dutch Central Bureau of Statistics [31] (Table 2).

The costs prices of health care practitioners consist of all costs directly and indirectly attributable to the unit (these are the costs of personnel, medical staff, material, medical apparatus, medical supporting departments, accommodation and overhead).

#### Cost-effectiveness measures

In the cost-effectiveness analyses the VAS, self-perceived WRULD and DASH-DLV are related to the health care costs. The QALY is related to the societal costs including productivity costs, in a cost-utility analysis.

### Data analysis

The expected improvement in pain in the PE group was set at 60% and for the RP group at 40%, implying a minimal clinical relevant difference of 20%, correlating with 20 mm difference on the horizontal VAS-scale. These expected improvements in pain were based on past clinical experience in our department of rehabilitation of the Maastricht University Hospital [11]. With an alpha of 0.05 and a 1-beta of 80% in total n = 94 computer screen-workers were needed to provide sufficient power to answer the research questions.

Data were analyzed by a blinded statistician using SPSS 13.0 for Windows (version 13.0; SPSS inc. Chicago, Ill.)

Data were checked for missing values and normality. Missing values have been replaced by mean imputation and by LOC-F (Last Observation Carried Forward) method. Each follow-up moment was analyzed separately and the analyses were carried out according to the intention to treat principle. Differences in baseline characteristics and baseline values of the outcome measures between PE and RP group were tested with an independent samples t-test (α = 0.05). In the event of significant differences between the two groups in baseline characteristics, adjustments were made in the statistical analyses [11].

The primary effectiveness measure, the horizontal VAS according to Jensen, has been analyzed at each follow-up moment by a t-test. Scores on DASH and EQ-5D questionnaires have been dealt with in the same way. The χ^2^-test has been used to analyze the answer on the question put dichotomously: "do you still experience non-specific WRULD complaints, yes or no".

The costs of both patient groups were compared by the bootstrapping method making use of confidence intervals in percentiles. By bootstrapping samples of the same size as the original data are drawn with replacement from the observed data [32]. In our study thousand bootstrap samples/replications were drawn.

The economic evaluation concerns cost-effectiveness and cost-utility analyses. The incremental cost-effectiveness ratios (ICER) are calculated based on the measured costs and outcome parameters. Health care costs are related to the medical outcome parameters and societal costs, including productivity costs are related to the QALY. Bootstrapping is performed and the simulated ratios indicate the uncertainty of the ICERs of the observed data. The ICERs resulting from bootstrapping are plotted on a cost-effectiveness acceptability curve (CEAC) indicating the probability for a range of ceiling ratios (society's maximum willingness to pay for one unit of effectiveness) that the cost-effectiveness of the PE treatment is acceptable [33].

Table 2 presents all cost prices with some items having a minimum and maximum cost price. Due to uncertainty concerning the cost price estimates two deterministic sensitivity analyses are performed [26]. These prices are varied simultaneously, once as minimum and once as maximum cost prices in the sensitivity analysis. In the sensitivity analysis with minimized cost prices, the productivity costs are based on 28.33 (28'20") contract hours divided over five working days a week. These contract hours are based on data of the CBS concerning the average working hours of the total working population in the Netherlands in 2004 [34]. This minimizes the productivity costs.

## Results

313 potential participants reacted or on the advertisements or were recruited by occupational physicians. Participants were selected and diagnosed between May 2003 and February 2005 and each participant had to complete a short questionnaire. The Saltsa-report [35] has been used as a guidebook to enable the correct diagnosis "early non-specific WRULD" by excluding potential participants with all kinds of specific WRULD [11].

**Figure 1:**
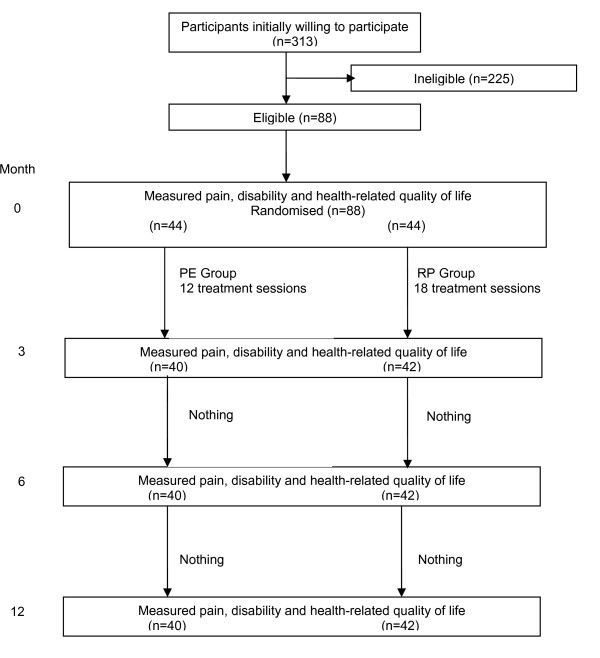
**Flow chart describing the routing of the participants through the trial**.

Finally 28% of these potential participants (i.e. 88 participants) have been included in this study, meeting the inclusion criteria and willing to participate. Information about the routing of the participants through the trial is presented in Figure 1.

Many potential participants had to be excluded for more than one reason[11].

Forty-four participants were randomized to each arm of the trial.

Both groups were comparable at baseline for nearly all variables except the score on the Pain Catastrophizing Scale (Table 3).

**Table 3:** Baseline characteristics of the PE group and the RP group

	PE group (n = 44)	RP group (n = 44)	p-value
Gender M:F	19:25	19:25	-
Education High:Low	29:15	30:14	-
Age (yr), mean (SD)	33.3 (7.7)	34.8 (7.7)	0.38
Pain (VAS, 0-10 cm), mean (SD)	2.9 (1.5)	2.6 (1.8)	0.40
Functional disability (Disabilities of Arm, Shoulder and Hand questionnaire) (0-100), mean (SD)	15.2 (10.3)	16.1 (12.3)	0.72
Quality of life (EQ-5D) (0.00-1.00)	0.83 (0.11)	0.86 (0.10)	0.32
Multidimensional Perfectionism Scale (29-145), mean (SD)	62.7 (16.4)	63.2 (18.7)	0.89
State Anxiety Inventory (20-80), mean (SD)	32.9 (8.9)	33.1 (10.6)	0.93
Trait Anxiety Inventory (20-80), mean (SD)	34.5 (9.9)	35.3 (9.8)	0.69
Self-reported fitness (9-45), mean (SD)	26.9 (2.8)	26.7 (2.1)	0.64
Fitness mark (1-10), mean (SD)	7.0 (1.1)	7.2 (1.5)	0.38
Job Stress Survey (0-81), mean (SD)	16.2 (10.7)	15.6 (10.0)	0.82
Pain Catastrophising Scale (0-52), mean (SD)	22.5 (6.6)	25.5 (6.3)	0.04
Duration of complaints < 6 weeks N = 16 > 6 weeks N = 72	8 36	8 36	
Working hours per week, mean (SD)	37.2 (10.7)	38.5 (6.3)	0.5

At baseline all data were available of the 88 participants. Between baseline assessment and three months assessment there were six drop outs for various reasons, four of the PE group and two of the RP group. Besides three non-compliant participants in the PE group, there was one participant who followed therapy, but resigned immediately after the therapy. One of the two non-compliants from the RP group wanted the other intervention after three treatment sessions, being the reason for his drop out.

After accounting for participants who stopped attending because they were free of complaints, compliance was 94% in the PE therapy group and 96% in the RP group [11].

### Effectiveness outcomes

Table 4 reports the mean scores of groups, group differences and 95% confidence intervals per outcome measure at baseline, three months, six months and one year after baseline. At three months, the RP group experienced significantly less pain as compared to the PE group, however this difference was not maintained at six and twelve months [11]. Otherwise no significant differences between the groups were observed. Also the QALY shows no significant differences between both groups.

**Table 4:** Mean scores (PE and RP) and group differences (95%CI) per outcome measure at baseline, 3 months, 6 months and 1 year after baseline

	PE (n = 44)	RP (n = 44)	Differences between groups
	*Mean (95%CI)*	*Mean (95%CI)*	*Mean (95% CI)*
VAS (10.0-0.0)*			
Baseline score	2.88 (2.43; 3.33)	2.59 (2.07; 3.11)	0.29 (-0.40; 0.99)
3 months	1.90 (1.35; 2.45)	1.13 (0.76; 1.51)	0.77 (0.09; 1.44)
6 months	1.32 (0.93; 1.72)	1.13 (0.76; 1.50)	0.19 (-0.36; 0.75)
1 year	1.41 (0.91; 1.91)	1.37 (0.91; 1.82)	0.04 (-0.64; 0.73)

**Self-perceived** **WRULD†**			
Baseline	100	100	0
3 months	68.18 (54.4; 81.9)	63.64 (49.4; 77.9)	4.5 (-14.9; 23.4)
6 months	47.73 (33.0; 62.5)	47.73 (33.0; 62.5)	0.00 (-20.4; 20.4)
1 year	43.18 (28.5; 57.8)	45.45 (30.7; 60.2)	-2.30 (-22.5; 18.1)

DASH (0-100)*			
Baseline score	15.23 (12.18; 18.27)	16.12 (12.47; 19.76)	- 0.89 (-5.71; 3.93)
3 months	10.98 (8.06; 13.91)	8.75 (5.89; 11.62)	2.23 (-1.92; 6.38)
6 months	9.94 (7.27; 12.62)	7.78 (4.93; 10.64)	2.16 (-1.81; 6.13)
1 year	9.33 (6.51; 12.15)	8.22 (5.19; 11.25)	1.11 (-3.09; 5.31)

EQ-5D (0.00-1.00)*			
Baseline score	0.83 (0.80; 0.87)	0.86 (0.83; 0.89)	- 0.02 (-0.07; 0.02)
3 months	0.89 (0.86; 0.92)	0.92 (0.88; 0.95)	- 0.03 (-0.07; 0.02)
6 months	0.92 (0.90; 0.95)	0.91 (0.89; 0.94)	0.01 (-0.03; 0.05)
1 year	0.91 (0.88; 0.95)	0.90 (0.87; 0.94)	0.01 (-0.04; 0.06)
**QALY (0.00-1.00)**	0.88 (0.86; 0.91)	0.87 (0.84; 0.90)	0.02 (-0.02; 0.06)

* T-test

† Expressed in % of patients with complaints; χ^2 ^test

### Health care utilization and sick leave

Table 5 presents data on consumption volumes, out of pocket payments and the sick leave due to WRULD during the follow-up period. Only a few patients reported additional utilization of (non-)health care resources and/or work absenteeism represented by productivity costs. Most of the PE therapy and RP sessions took place during the ten weeks intervention period.

**Table 5:** Mean and maximum consumption volume, out of pocket payments and sick leave per patient 1 year after baseline

Type of utilization [Unit of measurement]	PE (n = 44)		RP (n = 44)	
	**Mean**	Max.*	**Mean**	Max.*

***Volumes of care***				

GP care				

- Standard GP consult [no. visits]	0.60	6.00	0.54	6.00

- GP consult by phone [no. contacts]	0.08	1.75	0.18	6.00

- GP assistent [no. visits]	0.09	4.00	0.08	3.50

- GP repeat prescription [no. contacts]	0	0	0.04	1.75

Mensendieck/Cesar therapy (PE) [no. sessions]	18.00	38.00	0.05	2.00

Physiotherapy (RP) [no. sessions]	1.41	18.00	16.52	44.50

Ergo therapy [no. sessions]	0	0	0.05	2.00

Company doctor [no. visits]	0.05	2.00	0.09	4.00

Day treatment [no. sessions]	0	0	0.04	1.75

Psychology of primary care [no. sessions]	0	0	0.23	10.00

Policlinic consults				

- Radiology	0	0	0.05	2.00

- Orthopedics	0	0	0.29	12.75

- Specialist in general	0	0	0.12	5.25

Home care [no. hours a week]	0.12	5.25	< 0.01	0.06

***Out of pocket payments and sick leave***				

Devices hand/arm [costs €]	84.11	900.00	57.71	1668.80

Devices transport [costs €]	0.31	13.50	4.71	138.00

Other devices [costs €]	47.13	1256.19	126.99	2846.04

Medication [costs €]	1.10	20.88	0.40	17.12

Productivity costs [costs €]	316.80	13478.40	919.64	20160.00

* The minimum consumption and costs of all items is zero.

### Costs

Table 6 shows the mean costs (health care costs comprising costs for patient and family and productivity costs as well as societal costs) per patient group at baseline and mean cumulative (societal) costs per patient group after 3 months and one year. The upper and lower confidence limits in the table are the 2.5^th ^and 97.5^th ^percentile based on bootstrap replications.

**Table 6:** Mean costs per patient group at baseline and mean cumulative costs per patient group after 3 months and 1 year (95% CI)*

	PE (n = 44)			RP (n = 44)		
	Mean costs Bootstrapped mean costs (95% CI) *			Mean costs Bootstrapped mean costs (95% CI)*		

**Time elapsed**	**baseline**	**3 months**	**1 year**	**baseline**	**3 months**	**1 year**

**Health care costs**						
**- Treatment costs**	0 0 (0; 0)	583 584 (542; 615)	666 666 (600; 735)	0 0 (0; 0)	486 486 (441; 524)	563 565 (485; 651)
**- Other costs**	22 22 (10; 39)	6 6 (2; 11)	29 29 (12; 49)	23 23 (11; 38)	26 26 (8; 52)	125 124 (48; 228)
***Total health care costs***	*22* *22 (10; 41)*	*589* *589 (547; 620)*	*694* *693 (621; 764)*	*23* *23 (10; 39)*	*512* *512 (463; 559)*	*688* *684 (550; 839)*

**Costs for the patient and family**	36 35 (6; 80)	104 105 (42; 188)	164 166 (83; 268)	8 8 (3; 14)	107 106 (1; 243)	190 189 (33; 398)

**Productivity costs**	0 0 (0; 0)	0 0 (0; 0)	317 323 (0; 940)	0 0 (0; 0)	106 109 (0; 318)	920 913 (24; 2106)

***Societal costs***	*58* *59 (21; 110)*	*693* *694 (612; 786)*	*1176* *1152 (764; 1890)*	*31* *31 (15; 50)*	*725* *722 (528; 1017)*	*1797* *1817 (830; 3099)*

* The mean costs; the upper and lower confidence limits are the 2.5^th ^and 97.5^th ^percentile based on bootstrap replications

Table 7 shows the mean differences in costs between the two groups at baseline, after three months and one year. Also here the upper and the lower confidence limits are the 2.5^th ^and 97.5^th ^percentile based on bootstrap replications.

**Table 7:** Mean differences in costs between groups at baseline, after 3 months and 1 year (95% CI)*

	Mean difference		
	Bootstrapped mean difference (95% CI)*		
**Time elapsed**	**Baseline**	**3 months**	**1 year**

**Health care costs**			
**- Treatment costs**	0 0 (0; 0)	97 99 (42; 156)	103 101 (-4; 205)
**- Other costs**	-1 -1 (-21;19)	-20 -21 (-47; -1)	-97 -96 (-200; -19)
***Total health care costs***	*-1* *-1 (-20; 22)*	*77* *77 (11; 135)*	*6* *9 (-164; 168)*

**Costs for the patient and family**	28 28 (-2; 72)	-2 -1 (-150; 126)	-25 -23 (-252; 159)

**Productivity costs**	0 0 (0; 0)	-106 -109 (-318; 0)	-603 -590 (-1862, 521)

***Societal costs***	*28* *28 (-13; 81)*	*-31* *-29 (-341; 191)*	*-622* *-665 (-2087, 590)*

* The mean difference in costs between patient groups; the upper and lower confidence limits are the 2.5^th ^and 97.5^th ^percentile based on bootstrap replications

### Baseline

At baseline the health care costs and productivity costs are about the same. The costs for the patient and family and the societal costs have a rather small cost difference of €28 between the two patient groups (95% CI; -2, +72 respectively -13, +81).

### Follow-up

The PE group has higher treatment costs during the follow-up period compared to the RP group. Although other health care costs were lower in the PE group, the total health care costs indicate that the PE group has higher costs opposed to the RP group during the period after baseline. Concerning all other follow-up cost items the PE group is less costly than the RP group.

### Health care costs

During the intervention period the total health care costs of the PE group are higher (mean costs €589 PE vs. RP €512; incremental cost +€77; 95% CI +11, +135). There were no differences between both groups in costs over 1-year follow-up (mean costs €694 PE versus RP €688; incremental cost + €6; 95% CI -164; +168). The health care costs mainly consist of the costs of the treatments for PE therapy or RP during the ten weeks intervention period. Concerning the other health care costs, the PE group is less costly than the RP group with €29 versus €125 at one year after baseline (incremental cost at one year - €97, 95% CI; -200, -19) and €6 versus €26 just after the intervention period

### Costs for the patient and family

There were no differences between both groups in costs at three months respectively 1-year follow-up (incremental costs -€2; 95% CI; -150, +126 resp. -€25; 95% CI; -252, +159).

### Productivity costs

During the follow-up period only few patients (four patients in the RP group and two patients in the PE group) of both treatment groups had productivity loss due to their non-specific WRULD complaints. At one year after baseline the mean costs due to productivity loss were €317 in the PE group and €920 in the RP group (incremental cost at one year -€603; 95% CI; -1862, +521). At three months incremental cost was -€106; 95% CI; -318, 0.

### Societal costs

The mean societal costs one year after baseline are €1797 for the RP group, which is about €622 more (95% CI; -2087, +590) than the mean societal costs of €1176 of the PE therapy group. The difference between the mean health care and the mean societal costs per patient of the RP group in relation to the PE group is mainly attributable to the higher productivity costs in the RP group.

### Cost-effectiveness analysis

Because of a possible bias due to group differences at baseline (although not statistically different) in combination with small differences at follow-up regarding VAS-scores, DASH and EQ5D, the (Incremental Cost-Effectiveness Ratio) ICERs of the PE versus the RP treatment are calculated at three months and one year after baseline based on change scores of each group (see Table 8). Regarding all incremental costs and effects we calculated the upper and lower confidence limits of 2.5^th ^and 97.5^th ^percentile based on bootstrap replications.

**Table 8:** Mean differences in incremental effects/costs and incremental cost-effectiveness ratios of PE versus RP 3 months and 1 year after baseline (95% CI)*

Time elapsed	3 months			1 year		
	Incremental effect	Incremental costs	ICER #	Incremental effect	Incremental costs	ICER #
**Health care costs in relation to**						
**- VAS**	0.48 (-0.17; 1.12)	77 (11;135)	160.74 (-1185, 1924)	-0.25 (-0.99; 0.57)	6 (-164; 168)	-26.38 (I) (-2186, 2777)
**- Self-perceived WRULD**	-0.05 (-0.25, 0.16)	77 (11;135)	-1690.22 (I) (-4337, xxx)	0.02 (-0.18; 0.23)	6 (-164; 168)	285.65 (-4131, xxx)
**- DASH**	-3.14 (-7.22, 0.73)	77 (11;135)	-24.48 (I) (-204; 108)	-1.99 (-6.68; 2.20)	6 (-164; 168)	-3.27 (I) (-399; 327)

**Societal costs in relation to**						
**- QALY**				0.02 (-0.02, 0.06)	-622 (-2087, 590)	-33772.60(D) (-324027, 240226)

* The upper and lower confidence limits are the 2.5^th ^and 97.5^th ^percentile based on bootstrap replications

xxx = non-existent

(I): PE strategy is inferior compared to RP treatment

(D): PE strategy dominates RP treatment

Note 1: the above presented incremental costs and effects are based on change scores instead of the absolute costs and effects as presented in Table 4.

Note 2: # negative ICERs should be interpreted with caution.

**Figure 2:**
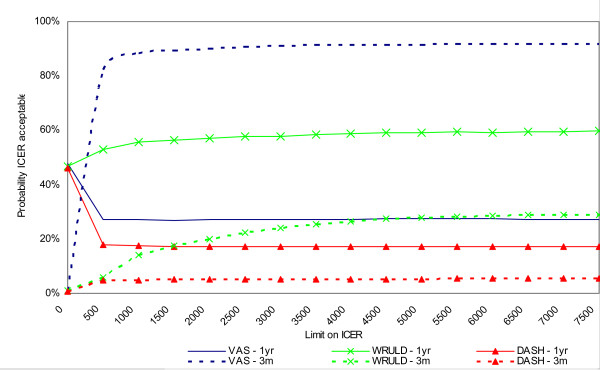
**Cost-effectiveness acceptability curve with health care costs**.

**Figure 3:**
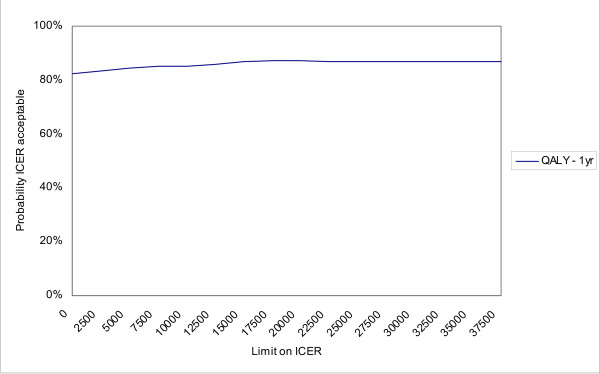
**Cost-effectiveness acceptability curve with societal costs**.

Negative ICERs should be interpreted with caution. These can indicate both dominance (higher effectiveness ànd lower costs) and inferiority (lower effectiveness ànd higher costs) of PE strategy over RP treatment; therefore we refer to Figures 2 and 3 for clarification (see further).

### Three months after baseline

Using health care costs the ICER for the VAS pain intensity at three months after baseline is about €161 per unit of improvement. This means that an additional amount of €161 is needed to achieve an improvement of one point on the VAS scale through Mensendieck/Cesar therapy as opposed to regular physiotherapy.

Concerning the three-month follow-up effectiveness measures self-perceived WRULD and DASH; the PE treatment is both more expensive and less effective as the RP. This indicates that the PE treatment is inferior opposed to the RP when evaluated from the self-perceived WRULD and DASH at a three-month follow-up period.

### One year after baseline

At one-year follow-up, the PE treatment is both more expensive (health care costs) and less effective regarding pain (VAS) and disability (DASH), indicating that the PE treatment is inferior opposed to the RP.

The ICER for self-perceived WRULD one year after baseline is about €286, meaning that an additional €286 is needed to achieve one more complaint-free patient through PE therapy.

The societal costs of the PE therapy group are lower compared to the RP group, while the treatment is more effective in terms of QALYs during the one-year period. Consequently the PE treatment is considered dominant from this societal perspective. The gain of an additional QALY through PE treatment implicates a cost saving of about €33.773. =

### Cost-effectiveness acceptability curve

Figure 2 presents the cost-effectiveness acceptability curves (CEACs) with the health care costs related to the effectiveness outcomes on the VAS, the self-perceived WRULD and the DASH at three months and one year after baseline. When the willingness to pay for an additional unit of effect on one of these outcome parameters is zero, the PE treatment tends towards inferiority. At three months after baseline there is a probability of only 1% that the ICER is acceptable at a ceiling of zero. At one year after baseline the probability that the PE treatment is cost-effective is about 46 to 48% for these three parameters. When the limit on the ICER is increased the PE treatment tends towards dominance concerning the change in VAS pain intensity achieved after three months (probability increases to 92%) and the number of patients with self-perceived WRULD-complaints after one year (probability increases to 60%). The other CEACs in Figure 2 still tend towards inferiority of the PE treatment, even when the willingness to pay increases. The cost utility analysis concerns the incremental QALY compared to the incremental societal costs during the one year follow-up period. As shown by Figure 3 the probability that the PE treatment is cost-effective is about 82 to 87%, depending on the ceiling ratio. From this perspective the PE treatment has a high probability to be cost-effective compared to RP treatment.

### Sensitivity analysis

The sensitivity analysis with maximized costs resulted in mean health care costs of €704 and societal costs of €1876 per RP patient compared to €697 respectively €1191 per PE therapy patient. In the sensitivity analysis with minimum costs, the mean health care costs decreased to €671 and societal costs decreased to €1507 per RP patient compared to €692 respectively €1073 per PE therapy patient. These sensitivity analyses only lead to small changes in the cost differences between both patient groups. The shape of the CEACs of the sensitivity analyses is comparable to the CEACs of the baseline analyses.

## Discussion

Little research has been done regarding cost-effectiveness in WRULD-patients.

One cost-effectiveness study [12] has been done among WRULD-patients with chronic complaints and one study is still running [13]. The study of Meijer (2006) shows that there is no difference in cost-effectiveness between two groups which were treated by multidisciplinary intervention respectively usual care.

This cost-effectiveness study, comprising WRULD-related health care costs including costs for the patient and family and productivity costs resulting in societal costs, is as a randomized controlled trial in computer screen-workers with early stages of non-specific WRULD the first of its kind. There was only a small rate of missing values in this research making the chance of bias low. The low percentage of dropout (less than 10%) was the reason to perform only an intention-to-treat analysis and no per-protocol analysis. Moreover this patient group concerns patients with early non-specific WRULD complaints of which is assumed that their dropout would not imply a significant impact on the results.

The results failed to show PE therapy according to Mensendieck/Cesar is more effective in computer screen-workers with early non-specific WRULD with respect to the effectiveness outcome measures (Table 4). In both groups there are small improvements over the one year follow-up period while after one year 55% of the participants reports to have no complaints any longer. The small effects on the outcome measures possibly can be explained by the fact that only patients with beginning complaints were included and therefore showed low scores on the scales. Despite the finding that there was no difference between groups we did not change the principle of conducting the cost-effectiveness analysis since our trial was based on the expectation of a difference in effectiveness between treatments [36]. Our study shows wide confidence intervals on both effectiveness and cost differences between the two treatment arms. Due to uncertainty surrounding our estimate of treatment effects arising from the negative results and small size of our trial, interpretation of the ICERs should be undertaking with caution [37].

**Figure 4:**
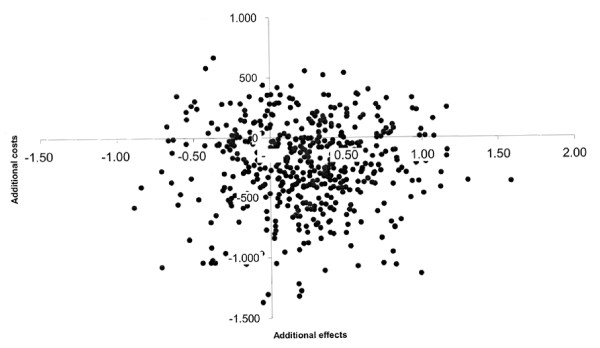
**Result of the bootstrap run based on the incremental costs per VAS: Incremental cost-effectiveness plane**.

Moreover, Figure 4 shows the results of the bootstrap run on the incremental costs per VAS. Given the fact that the bootstrapped ICERs are scattered around the origin, the confidence intervals of the ICERs presented in Table 8 are difficult to interpret.

Non-specific WRULD is 'work-related [16]. However, this research did not include a work-related effectiveness measure. Suffering from beginning complaints and according to the inclusion criteria, all participants were still at work at start of the treatment. Consequently "return-to-job" was not a useful effectiveness measure for this particular patient group.

Regarding cost-effectiveness, mean total health care costs including costs for the patient and family did not differ significantly between both groups at one year follow-up. This despite the fact that during the intervention period when most health care costs were made and mainly consisted of the costs of the treatments, these costs were higher in the PE group having one and a half hours more therapy compared to the RP group. On the other hand, productivity costs after one year were lower in the PE group. Productivity costs were based on 28.33 (28'20") contract hours divided over five working days a week [38]. However, our participants worked much more hours per week (ca. 38 hours per week). Productivity costs were calculated according to the friction cost approach, but this research did not take into account all of the possible productivity costs items from this approach. For example the productivity costs during the work due to decreased work performance called presenteeism [27] were not questioned in this research. These observations probably reflect an underestimation of the productivity costs measured in this study with possibly consequences for the outcome.

The mean societal costs one year after baseline were in favor of the PE therapy group, mainly attributable to the higher productivity costs in the RP group.

From the health care costs perspective at three months follow-up only with respect to the VAS the PE strategy had a high probability of acceptable cost-effectiveness. At one year follow-up the PE strategy had only regarding the self-perceived WRULD a- not the 60% exceeding- probability of acceptable cost-effectiveness.

Considering societal costs in relation to QALYs, the PE treatment had a probability of over 80% to be cost-effective over a wide range of cost-effectiveness ceiling ratios.

However, differences were marginal, possibly because this study only concerned computer screen-workers with early stages of non-specific WRULD and already no significant differences between the groups were found in the effectiveness of both therapies themselves.

As our preference concerns the societal perspective and the QALYs we tend to prescribe postural exercise therapy according to Mensendieck/Cesar for computer screen-workers with early stages of non-specific WRULD.

## Conclusion

In conclusion, although our trial failed to find significant differences in VAS, QALYs and ICERs based on VAS and QALYs at one-year follow-up, CEACs suggest that the postural exercise therapy according to Mensendieck/Cesar has a higher probability of being cost-effective compared to regular physiotherapy; however further research is required.

## List of abbreviations

WRULD: work-related upper limb disorders; VAS: visual analogous scale; DASH-DLV: disabilities arm, shoulder and hand - Dutch language version; EQ-5D: Euro-Qol - 5 dimensions; QALY: quality adjusted life year; PE: postural exercise therapy; RP: regular physiotherapy; JSS: Job Stress Survey; MPS-F: Multidimensional Perfectionism Scale of Frost; STAI: State-Trait Anxiety Inventory; PCS: Pain Catastrophizing Scale; GP: general practitioner; LOC-F: Last Observation Carried Forward; ICER: incremental cost-effectiveness ratio; CEAC: cost-effectiveness acceptability curve; CBS: Centraal Bureau van de Statistiek (Central Bureau of Statistics); CI: confidence interval.

## Competing interests

The authors declare that they have no competing interests.

## Authors' contributions

MVEB: first writer of all head chapters, interpretation of data. SG: acquisition of all the data for cost-effectiveness study and analyzing data. RdB: daily supervisor giving comments, interpretation of data. JS: final analyses and check of the completed article, final approval.
